# Metallothioneins in Inflammatory Bowel Diseases: Importance in Pathogenesis and Potential Therapy Target

**DOI:** 10.1155/2021/6665697

**Published:** 2021-04-24

**Authors:** Anna Socha-Banasiak, Patrycja Sputa-Grzegrzółka, Jędrzej Grzegrzółka, Krzysztof Pacześ, Piotr Dzięgiel, Beata Sordyl, Hanna Romanowicz, Elżbieta Czkwianianc

**Affiliations:** ^**1**^ Department of Gastroenterology, Allergology and Pediatrics, Polish Mother's Memorial Hospital-Research Institute, Lodz 93-338, Poland; ^2^Division of Anatomy, Department of Human Morphology and Embryology, Wroclaw Medical University, Wroclaw 50-367, Poland; ^3^Division of Histology and Embryology, Department of Human Morphology and Embryology, Wroclaw Medical University, Wroclaw 50-367, Poland; ^4^Department of Pathology, Polish Mother's Memorial Hospital-Research Institute, Lodz 93-338, Poland

## Abstract

Immunological disorders, increased oxidative stress, and damage to the epithelial barrier play an important role in the pathogenesis of inflammatory bowel diseases (IBDs). In the treatment of patients with Crohn's disease (CD) and ulcerative colitis (UC), it is increasingly common to use biological drugs that selectively affect individual components of the inflammatory cascade. However, administering the medicines currently available does not always result in obtaining and maintaining remission, and it may also lead to the development of resistance to a given agent over time. Metallothioneins (MTs) belong to the group of low molecular weight proteins, which, among others, regulate the inflammation and homeostasis of heavy metals as well as participating in the regulation of the intensity of oxidative stress. The results of the studies conducted so far do not clearly indicate the role of MTs in the process of inflammation in patients with IBD. However, there are reports that suggest the possibility of using MTs as a potential target in the treatment of this group of patients.

## 1. Introduction

According to the current state of knowledge, it is assumed that the pathogenesis of inflammatory bowel diseases (IBDs) is multifactorial. In the literature, there is an emphasis on the role of genetic predisposition, immune system disorders, environmental factors (including the type of delivery, the diet, and the exposure to tobacco smoke), modification of the composition of the intestinal microbiome, and abnormalities of the local immune response to commensal microbiota [1–4]. In patients with any form of IBD, local inflammatory infiltration is observed, accompanied by damage to the epithelial barrier, increased secretion of proinflammatory cytokines, increased oxidative stress, and disturbed apoptosis. One of the key proinflammatory cytokines associated with the development of IBD is TNF*α*, released mainly by monocytes, macrophages, and lymphocytes [5]. The increase in local TNF*α* synthesis goes along with a cascade of immune phenomena related to the excessive secretion of proinflammatory cytokines (IL-1a, IL-1b, IL-2, IL-6, IL-8, IL-12, IL-17, IL-23, and IFN), and a decrease in the synthesis of anti-inflammatory cytokines (IL-4, IL-10). This is also accompanied by the activation of other signaling pathways involving a Th17 response [6, 7]. In the case of Crohn's disease (CD), inflammation is mainly related to the activation of Th1 cells and increased production of IFNɣ and TNF*α*. Th1 cell stimulation is promoted by IL-12, IL-23, IL-15, IL-18, and IL-21. In ulcerative colitis (UC), the mechanism of the inflammatory response is less understood, but it is associated with, for instance, an impaired humoral response and an excessive production of IL-13, among others [8]. Metallothioneins (MTs) also participate in the inflammation pathophysiology in IBD patients. However, their role in regulating the inflammatory process in this group of patients has not been clearly established [9].

Currently, the aim of IBD therapy is to stop disease progression, eliminate the active inflammatory process, achieve regeneration, and maintain a proper function of the gastrointestinal mucosa [1]. IBD conservative treatment includes 5-ASA preparations, glucocorticoids, and immunosuppressants (thiopurines, methotrexate, cyclosporine, and tacrolimus), as well as an increasingly broad spectrum of biological drugs. The latter include monoclonal antibodies directed against TNF*α* (infliximab, adalimumab, certolizumab, and golimumab), integrins (vedolizumab, natalizumab), interleukins IL-12 and IL-23 (ustekinumab), and Janus kinase inhibitors (tofacitinib) [10]. At present, it seems beneficial to include immunomodulatory drugs, including biological agents, early into the treatment, which increases the chance of regression of the inflammatory lesions and of changing the natural course of the disease [11–14]. However, the aforementioned multifactorial pathogenesis, still not fully understood, often makes it difficult to choose the optimal treatment for a given patient [15]. Moreover, therapy with biological drugs is not always effective, which is confirmed by the results of studies that show that about 20% of patients do not respond to the administration of anti-TNF*α* preparations [16]. It should also be mentioned that long-term immunosuppressive treatment is associated with numerous side effects [17, 18]. Therefore, it is extremely important to search for new diagnostic methods and treatment options based on a thorough understanding of the processes that regulate the development of IBD, including impaired functions of the immune system and microbiota composition [19]. Investigating the molecular mechanisms underlying IBD and correlating them with clinical experiments may result in innovative methods of treating these chronic gastrointestinal diseases—so far incurable and constantly increasing in frequency.

## 2. Metallothioneins and Their Role in the Regulation of Inflammation

MTs, described in 1957 by Margoshes and Vallee, belong to the group of low molecular weight proteins (6–8 kDa), commonly found in the plant and animal kingdoms. Their presence was first found in the horse kidney cortex. Cysteine, a sulfur-containing amino acid, is the main building block of MTs. This ensures MTs a wealth of sulfhydryl groups (-SH) and metal ion binding [20–22]. MT isoforms isolated from various tissues differ slightly in their amino acid composition. The structure of MTs consists of alpha domains, four Cd ions, and beta domains, binding three metal ions: two Zn ions and one Cd ion. The alpha and beta domains are bound by a lysine dimer [23]. MTs participate in the processes of regulation of the homeostasis of elements, including Zn and Cu, but they can also lead to the detoxification of heavy metals (e.g., cadmium and copper). Another basic function of MTs is the removal of oxygen-free radicals—mainly the hydroxyl radical (˙OH) and the superoxide radical anion (O_2_^−^) [24, 25].

**Figure 1:**
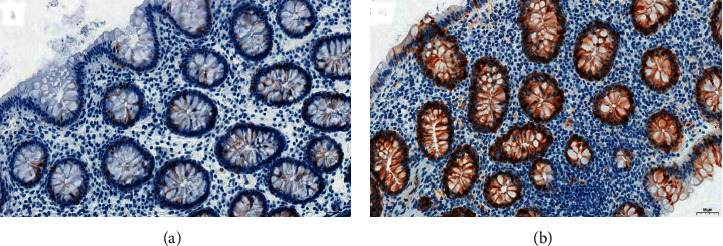
Analysis of immunohistochemical expression of metallothioneins I-II isoforms. (a) Low expression in colon epithelial cells and (b) high expression in colon epithelial cells and in some inflammatory cells in the tissue stroma. Magnification × 200. The immunohistochemical reactions were performed according to the IHC method described by Jankowska-Konsur et al. [30].

In humans, MTs are encoded by 19 genes located on the long arm of chromosome 16, but so far, only 11 types of functional MT proteins have been described (MT1A, MT1B, MT1E, MT1F, MT1G, MT1H, MT1M, MT1X, MT2A, MT3, and MT4) [25]. Moreover, four basic MT isoforms have been identified: MT-I, M-II, MT-III, and MT-IV. Isoforms MT-I and MT-II are common in both normal tissue and inflamed or neoplastic tissue. MT-III's expression has been found mainly in brain tissue, epithelial cells of the prostate, and kidneys, as well as in some neoplasms. The presence of the MT-IV isoform has been found, among others, in the stratified squamous epithelium and the upper gastrointestinal tract (Table 1) [26–29]. The analysis of immunohistochemical expression of metallothionein I-II isoforms in colon epithelial cells (own material) [30] was shown in Figure 1.

**Figure 2:**
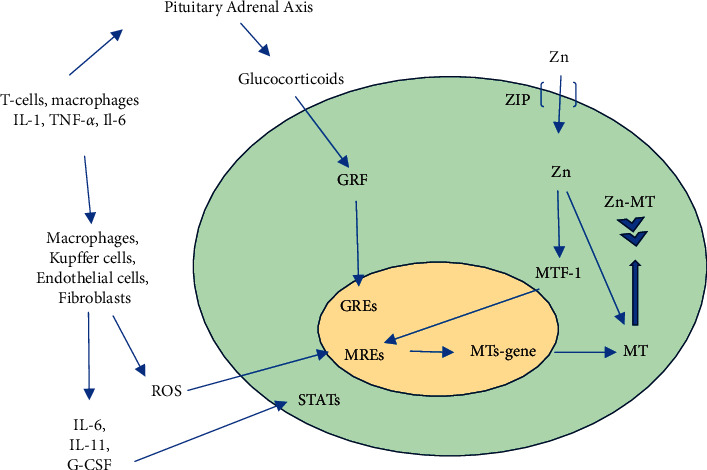
Hepatic MT gene regulation in inflammation. GREs: glucocorticoid responsive elements, GRF: cytoplasmic glucocorticoid receptor complex, MREs: metal response elements, MTF-1: metal transcription factor-1, STATs: signal transducers and activator proteins, and ROS: reactive oxygen species. Adapted from Coyle, et al. [37].

Local MTs expression regulation may depend, among other factors, on exposition to heavy metals, inflammation, and its related cytokine production and intensification of oxidative stress, and cancer [31–34]. An increase in MTs synthesis was observed in the case of inflammation caused by bacterial, viral, and fungal agents [34–36], as well as the use of glucocorticoids [25]. In case of inflammation, glucocorticoids lead to the increase in the intracellular labile Zn pool and promote activation of metal response elements (MREs) through a metal transcription factor (MTF-1). The immune cascade leads to the release of IL-6, IL-11 G-CSF, and reactive oxygen species (ROS) that interact with sites in the promotor region of the MT gene by inducing tyrosine phosphorylation of signal transducers and activator proteins (STATs) or metal response elements (MREs) (Figure 2) [37].

In studies in the animal model, it has been shown that, in mice with MT gene knockout, compared to wild-type specimens, both the respiratory and intraperitoneal administration of lipopolysaccharides cause significant tissue damage, which indicates anti-inflammatory properties of MTs, resulting, for instance, from the inhibition of proinflammatory cytokines [38, 39]. A significantly higher expression of cytokines such as IL-1, IL-6, macrophage inflammatory protein 2 (MIP-2), and keratinocyte chemoattractant (KC) was observed in the lungs, kidneys, liver, and blood of MT (-/-) mice compared to the control group [38, 39]. Other authors have shown that some proinflammatory cytokines, including TNF*α*, IL-1*α*, IL-1*β*, IL-6, and IFNɣ, are inducers for the MT-I and MT-II genes [34]. As previously mentioned, MTs are also important in antiviral defense [36]. An increase in the synthesis and an accumulation of MTs was observed in the nuclei of hepatocytes infected with the hepatitis C virus (HCV). The antiviral effect was enhanced by Zn administration, which induced the synthesis of MTs [40].

### 2.1. Role of Zn in the Regulation of Metallothionein Expression in Patients with IBD

Zn deficiency is often observed in patients with IBDs, especially those with CD [41]. This is a consequence of the lack of an adequate supply of this element as a result of an impaired appetite as well as hypoalbuminemia and malabsorption syndrome that may appear after the resection of a fragment of the small intestine [42]. So far, it has been demonstrated that Zn supplementation has a positive effect on the course of the disease, which was justified by the positive effect of this element in the tightness of the intestinal barrier, for which tight junctions (TJs), among others, are responsible [43]. The phenomenon described may also be related to the improvement of the epithelial repair functions, which was demonstrated in a study using intestinal epithelial cell lines. However, a positive effect was obtained after the cells were treated *in vitro* with physiological concentrations of Zn. The 10-fold higher dose of zinc led to cell damage and disorders of the repair functions [44]. Moreover, Zn sulphate has therapeutic effects on the cell oxidative damage by the increase of MT synthesis that was shown on the tongue tissues of rats [45].

In studies in the animal model, it has been shown that an increase in Zn consumption leads to an increase in MT synthesis in intestinal cells [46]. However, mice without MT expression accumulated lower amounts of Zn in the distal part of the gut despite a diet rich in this element compared to individuals from the control group (MT+/+) [47]. Other researchers confirmed in the mouse model the beneficial effects of excess dietary Zn on the prevention of colitis driven by the multifaceted roles of MTs [48]. Moreover, Zn supplementation may be a strong stimulant of MTs synthesis in the ischaemic tissue and reperfusion that was shown in the rat model [49]. It should be emphasized that Zn is recognized as one of the regulators of the functions of innate and acquired immunity. The participation of Zn has been shown in the process of activation of Th1 and B lymphocytes, as well as in the production of immunoglobulins. The function of macrophages is also positively regulated by the ions of this element, which ensures further NK cell activation, cytokine production, and phagocytosis [50]. Accordingly, in the case of Zn deficiency, an increased risk of infections due to bacteria, fungi, and viruses has been observed. Moreover, zinc deficiency is an important factor contributing to the immune aging process [51]. On the other hand, too high doses of this element may have a toxic effect on the cells of the immune system [52]. Studies have shown that MTs' participation in the immune response takes place, among others, by regulating the intracellular Zn pool [37]. In humans, about 10% of the entire proteome are Zn-binding enzymes or transcription factors, including proteins with the Cys4 and Cys2His2 zinc finger domains [53]. MTs, by influencing the accumulation of Zn in the cell or its release, are indirect regulators of the synthesis of Zn-dependent proteins important for the functioning of cells, such as Sp1, p53, or NF-*κ*B, which is a transcription factor for, among others, the IL-1*β*, TNF*α*, and ICAM-1 genes. This is of particular importance in the case of IBDs due to the participation of the aforementioned cytokines and adhesion molecules in the inflammatory process [54–56]. In an *in vitro* study, it was shown that Zn, by inducing the expression of the zinc finger protein A20, inhibits the activation of the nuclear factor kappa-light-chain-enhancer of activated B cells (NF-*κ*B), reducing the synthesis of proinflammatory cytokines, including TNF*α* [57], which is a key cytokine in the development of IBDs. Through zinc-binding, MTs may attenuate this phenomenon [54]. However, there are reports that indicate a negative influence of MTs on the expression of NF-*κ*B through interaction with TNF*α* [9, 58]. Intracellular Zn concentration depends on the activation of two families of Zn transporters: ZnT (SLC30) and Zip (SLC39). ZnT increases the influx of Zn ions into the cell and their storage in the cell organelles. In turn, Zip allows the influx of Zn into the cytoplasm [59]. It has been shown that the expression of these transporters can be regulated by proinflammatory cytokines [60].

At the same time, as mentioned earlier, Zn deficiency has a negative effect on the development and functions of both acquired and innate immunity that may promote autoimmune processes [60, 61]. In patients with Zn deficiency, a lower activity of thymulin has been observed. Thymulin is a hormone produced by the thymus necessary for the proper course of the maturation process of T helper (Th) lymphocytes; hence, a Zn deficiency leads to disturbances in the Th1/Th2 balance [62]. Additionally, it has been shown that the expression of Th1 profile cytokines (IL-2, IL-12, and IFNɣ) depends on the concentration of Zn ions [63]. It was observed in an *in vitro* study on human monocyte-derived macrophages (MDMs) and later confirmed *in vivo* in a mouse model that the stimulation of nucleotide-binding oligomerization domain-2 (NOD-2) receptors, belonging to the group of pattern recognition receptors (PRR), was associated with an increase in MT expression in MDM cells. As a consequence, increasing the intracellular Zn pool enhanced the clearance of bacteria via autophagy in macrophages [64]. It is worth noting that a faulty function of NOD-2 is associated with the development of CD [65]. Moreover, the aforementioned Zn membrane transporters (among others, Zip-8), whose expression is regulated by NF-*κ*B, participate in the inhibition of the inflammatory response through the regulation of the I*κ*B kinase (IKK), dependent on the concentration of Zn. This also confirms the potential participation of the element in the development of IBD [66].

### 2.2. Role of Metallothioneins in Antioxidant Processes in Patients with IBD

MTs, acting as scavengers of oxygen free radicals, play an important role in maintaining the antioxidant balance, which has been demonstrated in *in vitro* tests [67, 68]. Oxygen free radicals can be generated, among others, by UV radiation and X-rays. They can also be the result of reactions catalyzed by metals or simply be released by inflammatory cells, including macrophages and neutrophils during an inflammatory reaction [69]. Under physiological conditions, ROS are neutralized by enzymes such as superoxide dismutase (SOD), catalase (CAT), glutathione peroxidase (GPx), and glutathione reductase (GR). The inactivation of any of these enzymes weakens the body's antioxidant defense [70]. The glutathione-related antioxidant system also participates in the defense against ROS [71]. In the case of loss of the compensatory capacity of the system, ROS are not neutralized, which leads to lipid peroxidation, protein damage, and cell death. It has been demonstrated that, in comparison to healthy people, in patients with IBD, there is an increased local ROS activity that correlates with the disease activity and a decreased antioxidant barrier [72–74]. ROS damage the epithelial barrier by destroying the so-called tight connections, which increases the barrier permeability [75]. It was shown that lipid peroxidation, estimated by the malondialdehyde (MDA) concentration, was elevated in the plasma of IBD patients when compared to healthy individuals [76]. Moreover, MDA concentration was increased in both the inflamed CD and the inflamed UC mucosa. However, in CD, lipid peroxidation was independently associated with the concentration of MTs [77]. Luceri et al. demonstrated that, in the group of patients with CD, markers of oxidative stress such as advanced glycated end-products (AGEs) together with the specific receptor for advanced glycation end-products (RAGE) and advanced oxidation protein products (AOPP) may be pathogenic factors of the disease, but also potential targets of therapy [78]. Hamoude et al. observed in patients with UC (including those with dysplasia of the colon epithelium) a positive correlation between the concentration of AOPP in the serum and MTs [79]. The significant antioxidant properties of MTs have been demonstrated, for instance, in a study conducted by Miura et al. In the experiment, MTs reduced reactive forms of oxygen and inhibited the lipid peroxidation process in a significantly stronger way than reduced glutathione [80]. Therefore, overexpression of MTs in IBD patients may constitute an important form of defense against ROS generated during an intensified local inflammatory reaction [81, 82].

The local increase in MT expression is related not only to the presence of inflammation but also to decreased apoptosis and increased cell proliferation in, among others, Barrett's esophagus, tongue epithelium, and breast and colorectal cancer [83]. Rios et al. demonstrated in an animal model the antiapoptotic activity of MTs due, for example, to the protective effect on exposure to oxygen free radicals in rats subjected to spinal cord injuries [84]. By virtue of the likely different immune mechanisms observed in CU and CD and hence the different intensity of apoptosis in intestinal epithelial cells in both diseases, the expression of MTs should also differ between these two groups of patients [9]. Moreover, due to the influence of intestinal dysbiosis on an increased ROS activity, the antioxidant role of MTs may also play a significant role in the pathogenesis of UC and CD [73, 85].

### 2.3. MT Expression in IBD Patients

A review of the available literature shows individual studies in which MT expression was assessed both in an animal model of colitis and in IBD patients. Tsuji et al. conducted studies on a model of acute colitis in mice, and they demonstrated a positive role of MTs in preventing inflammation. In the experiments conducted by this group of researchers, after administration of the factor causing colitis, a significantly higher local increase in the synthesis of myeloperoxidase and proinflammatory cytokines (TNF*α*, IFN-*γ*, and IL-17) was observed in the group of mice with an MTI/II (-/-) knockout compared to wild-type specimens [86]. On the other hand, the results of the research conducted by Tran et al. indicate different conclusions. In an experiment carried out in mice with induced colitis, it was shown that animals with an MT (-/-) knockout had a lower disease severity than those MT (+/+), which suggests no preventive effect of MTs in the development of inflammation [87]. This is confirmed by the results of Devisscher et al., who observed that the blockade of extracellular MT pools may even lead to a reduction in colitis in mice [88].

In the studies conducted on human tissue, most authors showed a lower local MT expression in the intestinal epithelium of patients with IBD compared to the healthy population [89–92]. On the other hand, Bruwer et al. and Dooley et al. obtained the opposite results [93, 94]. MT expression in the intestinal mucosa was also compared in groups of patients with CD and UC, but the results did not show any statistically significant differences [91, 95]. The analyses also took into consideration different groups of drugs used in this group of patients. Elmes et al. [90] showed no significant effect of steroid therapy on MT expression, while Clarkson et al. [89] observed a higher local MT expression in patients treated with glucocorticoids. Attempts were also made to investigate the influence of inflammation on the intensity of MT expression. Devisscher et al., in an experimental model of colitis in mice, showed a higher local expression of MTs during the period of active inflammation than in remission [96]. Hamouda et al. assessed MTs as potential markers of dysplasia on the inflamed intestinal mucosa in patients with UC. However, no significant differences were observed between the concentration of MTs in the serum of patients with dysplasia and that of patients not suffering from it [79]. Genetic variants of MTs predisposing to the development of CD (New Zealand population) were also searched for, but no positive results were obtained [97].

Nevertheless, it should be emphasized that most of the studies presented were conducted in animal models or in small patient groups that did not even divide the patients into groups according to age and sex. Taking into account the different pathogenetic background of the disease in children and adults, it seems reasonable to take the age of the patients into consideration. Moreover, studies of the expression of MTs in IBD patients have so far been carried out with the use of many different techniques (immunohistochemistry, radioimmunological methods, and mRNA assessment), which, bearing in mind the limitations of each method, make it difficult to draw unambiguous conclusions due to the lack of repeated observations by independent researchers.

### 2.4. MTs as Potential Targets for IBD Treatment

As previously mentioned, the use of modern methods for treating patients with inflammatory bowel diseases, including biological drugs, does not always guarantee to achieve the remission of the disease. Researchers are constantly looking for new diagnostic markers and therapeutic targets, which should be aimed directly at inhibiting the development of the inflammatory process, based on a deeper understanding of the mechanism and supported by molecular biology methods [55].

The reports presented by Devisscher et al. suggest that MTs may be one of those therapeutic targets in patients with IBD due to their participation in the inflammatory process. This concept was based on the possibility of blocking the extracellular MT pool [88]. As described above, the intracellular MTs can act as a reservoir of essential heavy metals, a scavenger of reactive oxygen and nitrogen species, and play a protective role against inflammation [98]. On the other hand, the presence of MTs outside the cells is the result of the influence of stressors and cellular damage and may be observed in various conditions, including autoimmune diseases. Extracellular MTs may promote the migration of leukocytes to the place of inflammation [98, 99]. Thus, MT blockage may be effective in patients expressing elevated extracellular levels of MTs [55]. Studies in a mouse model with induced intestinal inflammation showed that MT (-/-) mice experienced a less intense inflammation (assessed on the basis of survival, weight loss, intestinal length, and intensity of local inflammatory cell infiltration) than wild-type specimens. Moreover, the authors showed that blocking the extracellular MT pool through the use of the appropriate antibodies inhibits the development of inflammatory infiltrate composed of macrophages. Additionally, with the use of single-photon emission tomography, after the administration of radioactive anti-MT antibodies to mice, they observed an antibody accumulation in the large intestine and a decrease in the signal during the period of clinical improvement. In a study on human tissue from patients with colitis, the researchers also showed inflammatory infiltrates composed of MT-expressing cells, which correlated with the severity of the inflammation [88].

Dostie et al. suggest that MT blockade with the use of monoclonal antibodies may be one of the methods for treating CD and UC patients in the future [55]. This concept is based on the results of studies that indicate MTs' function of stimulating the inflammatory process, regardless of whether these MTs are released from the cell or administered exogenously [88, 100]. This approach would have the advantage of blocking the extracellular pool of TMs released during tissue injury, which is when MTs act as a chemotactic factor for the inflammatory cells. The potential inhibition of the extracellular activity of MTs, for instance with the use of biological therapy, could prove effective in a group of patients diagnosed with MT overexpression. Due to the lack of activity in the intracellular MT pool, the activity of these proteins would not be limited, for example, in antioxidant processes. The aforementioned therapy could reduce the side effects observed when administering other biological preparations, and it would increase the treatment success rate in this group of patients [55]. However, it should be noted that any potential therapy should be approached with caution. The majority of cited studies were performed on cells or animal models, which is a significant limitation. Taking into account that the role of MTs in the pathogenesis of colitis is uncertain, the studies on representative groups, using human tissues, especially in different stages of inflammation, are necessary to discuss the role of MTs in the UC and CD pathogenesis and the potential goal of treatment. Moreover, it should be emphasized that the potential use of monoclonal antibodies in the future may probably be addressed only to the subjects expressing elevated extracellular levels of MTs.

## 3. Conclusions

The role of MTs in the inflammatory process in patients with IBDs has not been clearly defined so far. The regulation of the homeostasis of Zn ions and the antioxidant defense suggest a potential role of MTs in protecting cells against factors that can stimulate inflammation. However, local stimulation of the inflammatory process has been demonstrated both when cells release MTs and when MTs are administered exogenously. This discrepancy encourages the continuation of research in this area, especially with the use of human tissue and taking into account the clinicopathological data as well as the differentiation of patients with UC and CD. If the proinflammatory effect of MTs in IBDs is confirmed, the possibility of inducing the extracellular blockade of these proteins seems to be a promising solution in the treatment of this group of patients.

## Figures and Tables

**Table 1:** Occurrence of different isoforms of human metallothioneins (MTs).

MT-I/II	Nucleated cells of normal tissue and inflamed or neoplastic tissue, CNS glial cells, interstitial fluids, and blood plasma (in free form)
MT-III	Neurons, renal tubular cells, certain tumor cells
MT-IV	Squamous epithelial cells

CNS: central nervous system.

## Data Availability

The data used to support the findings of this study can be provided by the corresponding author upon request.
